# Fe_3_O_4_ Nanoparticles Attenuated *Salmonella* Infection in Chicken Liver Through Reactive Oxygen and Autophagy via PI3K/Akt/mTOR Signaling

**DOI:** 10.3389/fphys.2019.01580

**Published:** 2020-01-17

**Authors:** Yiru Shen, Yunqi Xiao, Shan Zhang, Shu Wu, Lizeng Gao, Shourong Shi

**Affiliations:** ^1^Poultry Institute, Chinese Academy of Agriculture Science, Yangzhou, China; ^2^Institute of Effective Evaluation of Feed and Feed Additive (Poultry Institute), Ministry of Agriculture, Yangzhou, China; ^3^Jiangsu Key Laboratory of Experimental & Translational Non-coding RNA Research, Institute of Translational Medicine, School of Medicine, Yangzhou University, Yangzhou, China; ^4^Jiangsu Co-Innovation Center for Prevention and Control of Important Animal Infectious Diseases and Zoonoses, Yangzhou, China

**Keywords:** Fe_3_O_4_ nanoparticle, *Salmonella* Enteritidis, chicken, reactive oxygen, autophagy

## Abstract

Recently nanomaterials have received substantial attention in biotechnology areas for their innovative properties in physical and chemical function. One of the most arrestive properties of nanomaterials that has been reported is their bacteriostatic activity. Our previous research found that Fe_3_O_4_ magnetic nanoparticles (Fe_3_O_4_-NPs) could effectively reduce the viability of intracellular *Salmonella* Enteritidis in chicken cells. There is an essential need to explore whether the bacteriostatic activity of Fe_3_O_4_-NPs is available *in vivo*. As an extension of this research, we conducted the present study to investigate the potential effect of Fe_3_O_4_-NPs used for *S*. Enteritidis control in chickens and to extensively investigate the underlying mechanisms in the process. The overall study included the evaluation of pathological sections, antioxidant status, inflammation, and the autophagy status of chicken liver, including the signaling pathway involved in the process. Results indicated that Fe_3_O_4_-NPs pretreatment can effectively inhibit the invasion of *S*. Enteritidis in chicken liver. Fe_3_O_4_-NPs pretreatment significantly increased reactive oxygen species (ROS) generation in chickens, including antioxidant enzyme activities. *S.* Enteritidis infection significantly increased the protein expression of the autophagy marker LC3. Additionally, the inflammation response and pathological changes caused by *S*. Enteritidis infection were alleviated by Fe_3_O_4_-NPs pretreatment. Phosphorylated mTOR was significantly increased in *S.* Enteritidis infected chickens, but showed no difference in chickens pretreated with Fe_3_O_4_-NPs. In summary, the results demonstrated that ROS and autophagy were involved in the inhibition of *S*. Enteritidis in chickens by Fe_3_O_4_-NPs pretreatment. The redox balance and inflammation response appeared normal in the process, as did the expression of the PI3K/Akt/mTOR signaling pathways. Taken together, our research demonstrate that the bacteriostatic activity of Fe_3_O_4_-NPs in chickens is avaliable and safe, which can be an alternative to antibiotics for bacterial inhibition in poultry industry.

## Introduction

*Salmonella*, one of the most widely spread pathogenic bacteria that infected in humans, shows great threat to people in hospital and community settings (Scallan et al., 2011). Large number of illnesses and deaths in humans have been reported to be caused by *Salmonella* infections annually worldwide (Hannemann and Galán, 2017). The most important routes of transmission of *Salmonella* to humans are poultry meat and eggs. *Salmonella* control in poultry infections has proven to be a universal topic of research in recent years. *Salmonella* Enteritidis, one of the most common serovars isolated from poultry (World Health Organization [WHO], 2018), is considered to survive in different cells (Haraga et al., 2008). Alarmingly, *S*. Enteritidis was reported to have multidrug resistance to several antibiotics because of the excessive use of antibiotics in animal husbandry. Hence the urgent task is now to discover novel and effective ways to treat infectious disease to protect both animals and humans.

Nanomaterials have received enormous attention for the creation of novel methods in biotechnology areas for their innovative properties in physical and chemical function (Sarkar and Sil, 2014; Gao and Yan, 2016). Under physiological conditions with no other enzymes and catalysts, nanomaterials can catalyze multiple bioreactors due to their intrinsic properties (Gao et al., 2007; He et al., 2014; Ragg et al., 2016). Nanozymes are classified to three classes until now by their enzyme-like properties: metal oxide activity, metal activity and carbon activity. Fe_3_O_4_ magnetic nanoparticles (Fe_3_O_4_-NPs), a common nanozyme that had metal oxide activity, demonstrate peroxidase-like activity or catalase-like activity in the mediums of different pH values (Wei and Wang, 2013). Importantly, Fe_3_O_4_-NPs is reported to inhibit the spread of multiple bacteria, such as *Escherichia coli* (Zhang et al., 2013), methicillin-resistant *Staphylococcus aureus* (Pan et al., 2016) and *Streptococcus mutans* (Gao et al., 2016).

Our previous work also found that Fe_3_O_4_-NPs could effectively reduce the viability of intracellular *S.* Enteritidis in chicken liver hepatocellular carcinoma cell line (LMH). Fe_3_O_4_-NPs is considered to be an alternative for the inhibition of chicken *S*. Enteritidis infections. However, little is known about the effect of Fe_3_O_4_-NPs *in vivo*. The potential role of Fe_3_O_4_-NPs in chickens are crucial, it would be necessary to discover the changes involved with these nanoparticles. Therefore, as part of an extended program of Fe_3_O_4_-NPs research, the aim of the present study was to evaluate the effect of Fe_3_O_4_-NPs in chickens infected with *S*. Enteritidis. The overall study included the determination of pathological sections, ROS levels, antioxidant status, the inflammation and autophagy status of chicken liver, and the PI3K/Akt/mTOR signaling pathway in the procedure. We are hoping the overall results of this study would present the potential effect of Fe_3_O_4_-NPs in chickens, which will be important evidence for the use of antibiotic alternatives for bacterial inhibition in chickens.

## Materials and Methods

### Preparation and Characterization of Fe_3_O_4_-NPs

Fe_3_O_4_ magnetic nanoparticles were prepared by the hydrothermal method with FeCl_3_ and NaAc.3H_2_O as raw materials. After fully dissolving in glycol or a mixture of glycol and diethylene glycol, they were transformed in a 50 mL teflon-sealed pressure-cooker and heated at 200°C for 12 h. The reactants were then cooled to ambient temperature and washed three times with water and ethanol each. The dry operation at 60°C for 6 h of reactants were under vacuum. The morphology, particle size and size distribution of Fe_3_O_4_-NPs were measured using scanning electronic microscopy (SEM, S-4800, Japan) and transmission electron microscopy (TEM, Tecnai G2 F30 S-TWIN, America). The diameter of Fe_3_O_4_-NPs in dispersion was determined using the dynamic light scattering (DLS) technique (Nano ZS90, United Kingdom). A laser instrument (Nano ZS90, United Kingdom) was used to measure the zeta potential of Fe_3_O_4_-NPs.

### Experimental Design

All experiments were conducted in accordance with the Regulations of the Experimental Animal Administration issued by the State Committee of Science and Technology of the People’s Republic of China. Three hundred and fifteen 1-day-old healthy specific pathogen-free (SPF) White Leghorn chickens were brought from Beijing Merial Vital Laboratory Animal Technology Co., Ltd. (Beijing, China) and weighed. Two hundred and forty chickens, with initial body weights of 42.15 ± 1.55 g, were equivalently weight-distributed into four groups: (1) control (denoted as C), (2) control + Fe_3_O_4_-NPs (denoted as N), (3) *S*. Enteritidis infected group (denoted as S), and (4) *S*. Enteritidis infected + Fe_3_O_4_-NPs (denoted as SN). Our previous study showed that Fe_3_O_4_-NPs can inhibit the planktonic *salmonella* in the culture medium. The direct exposure of *salmonella* to Fe_3_O_4_-NPs in the chicken’s intestine may influence the construction of the infection model in chickens. To avoid this, two different routes were chosen in this trial. Fe_3_O_4_-NPs were administered orally as the additives, while the infected chicken models were conducted with *salmonella* injection. The administration dose of Fe_3_O_4_-NPs was referred to the results of pilot experiments (Supplementary Figure S1). For all Fe_3_O_4_-NPs treatment group, birds at the ages of 2, 4, and 6 days-old were administered orally 50 mg/kg Fe_3_O_4_-NPs. For all *S*. Enteritidis infected group, birds at the ages of 7 days-old were administered with 0.1 mL of *S*. Enteritidis (1 × 10^8^ CFU/mL) by injection. Birds were housed groups individually with *ad libitum* to basal diet in cages. The diet of the birds was formulated according to NRC (1994) nutrient requirements without antibiotics and were negative for *S*. Enteritidis. The nutrient composition is shown in Table 1. This experiment was repeated twice independently.

**TABLE 1:** Diet composition and nutrient levels during the experiment (dry basis).

**Items**	**%**
**Ingredients**	
Corn	63.44
Soybean meal (46%)	31.80
Soybean oil	0.70
NaCl	0.30
Calcium hydrogen phosphate	1.50
Limestone	1.57
Methionine	0.20
L-lysine (hydrochloride)	0.11
Mineral premix^1^	0.20
Vitamin premix^2^	0.03
Phytase	0.15
Total	100.00
**Energy and nutrient^3^ composition**	
ME (kcal/kg)	2953.80
CP	21.00
Ca	1.00
Lys	1.11
Met	0.49
Met+Cys	0.81
Total phosphorus	0.58
Non-phytate phosphorous	0.45

*^1^The mineral premix provides the following per kg of diet: Fe, 80 mg; Cu, 16 mg; Mn, 120 mg; Zn, 110 mg; Se, 0.3 mg; I, 1.5 mg; and Co, 0.5 mg. ^2^The vitamin premix provides the following per kg of diet: Vitamin A (retinyl acetate), 15000 IU; Vitamin D3 (cholecalciferol), 3600 IU; Vitamin E (DL-α-tocopheryl acetate), 30 IU; Vitamin K, 3 mg; Vitamin B1, 2.7 mg; Vitamin B2, 9.6 mg; Vitamin B6, 3.75 mg; VB12, 0.03 mg; D-pantothenic acid, 14.1 mg; niacin, 45 mg; folic acid, 1.5 mg; and D-biotin, 0.15 mg. ^3^The nutrient levels were calculated values.*

### Liver Collection

Twenty four hours post the *S*. Enteritidis infection, birds from 4 groups were randomly selected for *S*. Enteritidis culture only (*n* = 15). 48 h post the *S*. Enteritidis infection, birds from 4 groups were randomly selected for multiple determinations(*n* = 15). The left side of the livers was removed, minced and snap-frozen before the storage at −80°C for protein and mRNA determination. Part of the right side of the liver was immediately fixed in a 10% formaldehyde solution for histology determination. The rest of the liver was collected for *S*. Enteritidis culture and other measurements.

### *Salmonella* Culture of Liver From Infected Chicken

Before the *salmonella* culture, liver samples were weighed, and homogenized in PBS with homogenizer from NingBo Scientz Biotechnology Co., Ltd. (Ningbo, China). Liver homogenates were then cultured on xylose lysine desoxycholate (XLD) agar plates (Qingdao Hope Bio Technology Co., Ltd., China) overnight at 37°C.

### Histology

The method of histology analysis was according to a study reported before (Desmidt et al., 1997). Liver samples from the same part of each birds were stored in formaldehyde solution(*n* = 6). Before the hematoxylin and eosin (H&E) staining for elastin, the liver samples were manipulated into sections with a depth of 5 μm, which was processed in paraffin. H&E stained paraffin sections were viewed under a bright field on an automatic image analyzer with Motic 3.0 software (BH2, Olympus, Japan). Liver samples were embedded in Epon 812 epoxy resin. Ultra-thin sections (about 70 nm) were cut and double stained with uranyl acetate and citromalic acid lead, and then observed using a CM100 transmission electron microscope (Philips) at 80 kV.

### Antioxidant and Peroxidation Activity

From the livers collected at the end of the study, the activity assay of malondialdehyde(MDA) superoxide dismutase (SOD), glutathione peroxidase (GSH-Px), and catalase (CAT) were conducted using the kits from Nanjing Jiancheng Institute of Bioengineering (Nanjing, China). Liver samples were homogenized in different volume of PBS to give the enzyme activity in the linear ranges, which were processed with pure enzymes.

### Real-Time PCR

From the livers collected at the end of the study, the expression of interferon-α (IFN-α), tumor necrosis factor (TNF-α), interleukin-6 (IL-6) and insulin-like growth factor 1 (IGF-1) were assayed using real-time PCR methods. Total RNA was extracted from frozen liver samples (Thermo Fisher Scientific, Waltham, MA, United States) and reverse-transcribed using 5 × PrimeScript RT Synthesis Kit (TaKaRa, Tokyo, Japan). The qPCR was performed using SYBR Green Real-time PCR Synthesis Kit (Qiagen, Hilden, Germany). The whole reaction was performed in a StepOnePlus Real-Time PCR System (Thermo Fisher Scientific, Waltham, MA, United States). The primers used are listed in Table 2. The band densities of target genes were normalized with those of β-actin. The relative abundances of target genes was calculated according to the 2^–ΔΔ*CT*^ method.

**TABLE 2:** Primer sequences used for real-time PCR and the GenBank number of the PCR products.

**Primer**	**Sequences (5′ to 3′)^2^**	**GenBank No.**
IFN-α^1^	F-GGACATGGCTCCCACACTAC R-GGCTGCTGAGGATTTTGAAGA	XM-004937097.1
TNF-α^1^	F-AATTTGCAGGCTGTTTCTGC R-TATGAAGGTGGTGCAGATGG	NM-204267
IL-6^1^	F-AGGACGAGATGTGCAAGAAGTTC R-TTGGGCAGGTTGAGGTTGTT	NM-204628.1
IGF-1^1^	F-TGTACTGTGCTCCAATAAAGC R-CTGTTTCCTGTGTTCCCTCTACTTG	NM-416323
β-actin	F-GAGAAATTGTGCGTGACATCA R-CCTGAACCTCTCATTGCCA	NM-205518

*^1^Abbreviations: IFN-α, interferon-α; TNF-α, tumor necrosis factor; IL-6, interleukin-6; IGF-I, insulin-like growth factor 1. ^2^F, forward primer, R, reverse primer.*

### Western Blotting

From the livers collected at the end of the study, the concentrations of microtubule-associated protein light chain 3(LC3), sequestosome 1(p62), protein kinase B (Akt), phosphorylation Akt (Ser 473), mammalian target of rapamycin (mTOR) and phosphorylation mTOR (Ser 2448) were assayed using western blotting methods. Liver proteins were extracted using RIPA buffer and bicinchoninicacid (BCA) Protein Assay Kit (23225, ThermoFisher Scientific, Waltham, MA, United States) was used to assay the concentrations. The primary antibodies were listed as bellows: rabbit anti-LC3B (1:850, Sigma, United States), p62 (1:1000, Sigma, United States), mTOR (1:1000, CST, United States), mTOR 2448 (1:1000, CST, United States), Akt (1:1000, CST, United States), Akt 473 (1:12000, CST, United States), β-actin (1:2000, Abcam, United States). The visualization and detection of the target proteins were performed in the Bio-Rad Chemidoc^TM^ XRS + (Bio-Rad, United States). The concentrations of the target proteins were analyzed using the Image Lab software (Bio-Rad, United States). The band densities of target protein were normalized with those of β-actin.

### Determination of ROS

Liver samples were weighed, and homogenized in PBS. The supernatant of liver were cultured with dichlorodi drofluorescein diacetate (DCFH-DA, Applygen Technologies Co., Ltd., Beijing, China) for 30 min. 2′, 7′-dichlorofluorescein (DCF), the reaction product of DCFH-DA, were assayed using the spectrofluorometer (722N, Shanghai precision Co., Ltd., China).

### Statistical Analysis

The statistical significance was assessed with one-way analyses of variance (ANOVA) test or independent-sample *T*-test. All the experiment data are expressed as the mean ±standard error of the mean (SEM). *P* < 0.05 was regarded as statistically significant. Different letters indicate statistically significant differences.

## Results

### Fe_3_O_4_-NPs Characterization

**FIGURE 1:**
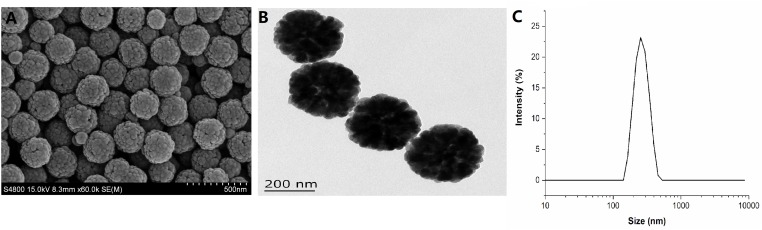
Characterization of Fe_3_O_4_ magnetic nanoparticles. **(A)** Representative scanning electronic microscopy (SEM) images of Fe_3_O_4_-NPs (scale bar = 500 nm). **(B)** Representative transmission electron microscopy (TEM) images of Fe_3_O_4_-NPs (scale bar = 200 nm). **(C)** Dynamic light scattering (DLS) of Fe_3_O_4_-NPs.

The morphology, particle size and size distribution of Fe_3_O_4_-NPs were measured using SEM and TEM (Figures 1A,B). The diameter of Fe_3_O_4_-NPs in dispersion was determined using the DLS technique (Figure 1C). Micrographs obtained by SEM (Figure 1A) and TEM (Figure 1B) showed that the nanoparticles were near-spherical and uniform in shape. The hydrodynamic diameters of the Fe_3_O_4_-NPs were measured by DLS. As shown in Figure 1C, the average hydrodynamic diameters of Fe_3_O_4_-NPs were 200 ± 6.79 nm, which was in good agreement with the TEM result. The zeta potentials were +20.3 ± 1.6 mV, indicating that the nanoparticles were positively charged, which is beneficial for later absorption by chickens. Results of this study revealed that the characteristic of Fe_3_O_4_-NPs showed stable and disperse, which were suitable for subsequent experiments. Micrographs of liver obtained by TEM (Supplementary Figure S2) showed that orally administrated Fe_3_O_4_-NPs were delivered to liver successfully.

### Fe_3_O_4_-NPs Inhibited *S.* Enteritidis Infection

**FIGURE 2:**
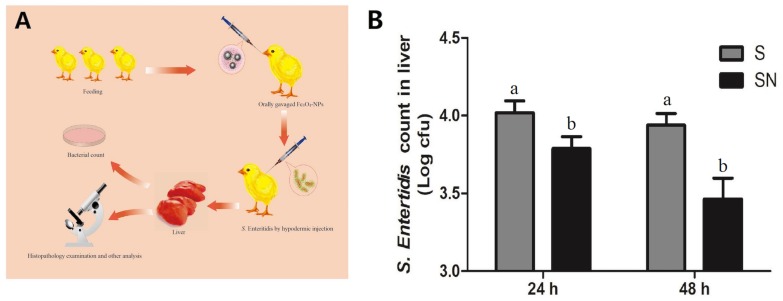
The inhibitory effects of Fe_3_O_4_-NPs on *Salmonella enteritidis* in chickens. Chickens were treated with control (C), Fe_3_O_4_-NPs alone (N), or infected with *S*. Enteritidis in the absence (S) or presence (SN) of Fe_3_O_4_-NPs. **(A)** Schematic illustration of the animal experiment. **(B)** Bacterial inhibition rates of Fe_3_O_4_-NPs at different times in livers of *S*. Enteritidis infected chickens. Values represent the mean ±SEM. Different letters indicate statistically significant differences.

A schematic illustration of the animal experiment is shown in Figure 2A. Chickens received gavages with Fe_3_O_4_-NPs were subcutaneously challenged with *S*. Enteritidis. Livers samples were collected for pathology examination and later analyses. The calculation and statistical results of *S.* Enteritidis counts in liver at 24 and 48 h post the injection are shown in Figure 2B. The *S*. Enteritidis counts of liver samples in Fe_3_O_4_-NPs-treated birds challenged with *S*. Enteritidis (group SN) were significant lower than that in birds infected with *S*. Enteritidis (group S) both in 24 and 48 h post the challenge. The inhibition rates in group SN were 36.77 and 66.42% at 24 and 48 h post injection, respectively, compared with group S. Our results confirmed that the Fe_3_O_4_-NPs can effectively inhibit *S*. Enteritidis infection in chicken liver.

### *S.* Enteritidis Induced Pathological Changes, Which Were Attenuated by Fe_3_O_4_-NPs

**FIGURE 3:**
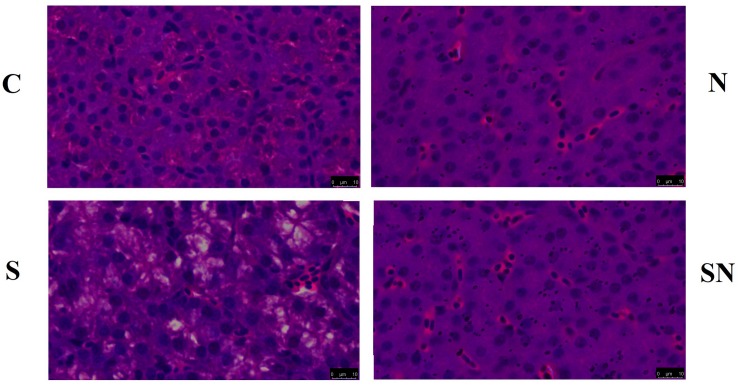
Representative photomicrographs of liver histological section of chickens exposed to Fe_3_O_4_-NPs in the absence or presence of *S*. Enteritidis administration (scale bar = 10 μm).

Results of the liver histopathology examination are shown in Figure 3. Birds in group C showed normal tissue with little vacuoles and intense structure, while birds in group N showed a similar structure with clustered Fe_3_O_4_-NPs distributed evenly in the interstitial space. Livers of birds in group S showed more vacuoles with widened interstitial space compared with birds in the other groups. Birds in group SN showed a similar structure to normal tissues with little vacuoles and evenly distributed Fe_3_O_4_-NPs. Our results confirmed that the liver pathological changes caused by *S*. Enteritidis infection was significantly mitigated in Fe_3_O_4_-NPs treatment groups.

### Fe_3_O_4_-NPs Increased ROS Production

**FIGURE 4:**
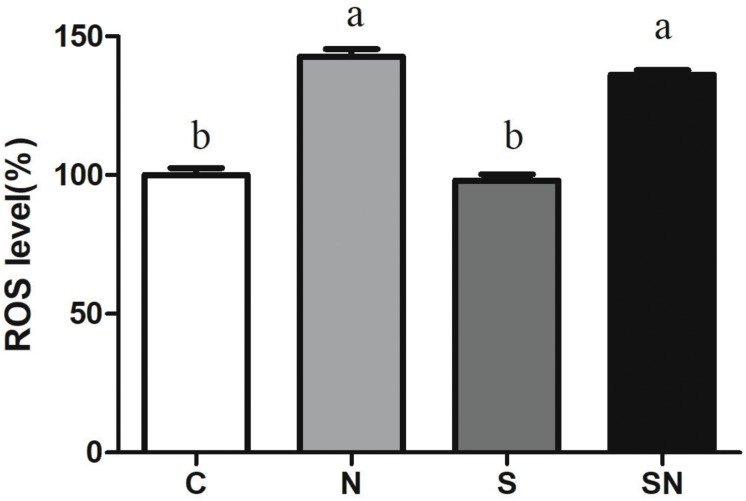
Effect of Fe_3_O_4_-NPs on ROS production in the livers of chickens exposed to Fe_3_O_4_-NPs in the absence or presence of *S*. Enteritidis administration. Values represent the mean ±SEM. Different letters indicate statistically significant differences.

Reactive oxygen species levels in the four groups are shown in Figure 4. Fe_3_O_4_-NPs exposed chickens in group N and SN showed significantly increased ROS levels compared with chickens in the control (group C) and *S*. Enteritidis infection groups (group S). *S.* Enteritidis infection alone had no significant effect on ROS production relative to the control group.

### Fe_3_O_4_-NPs Increased Antioxidant Enzyme Activities

**FIGURE 5:**
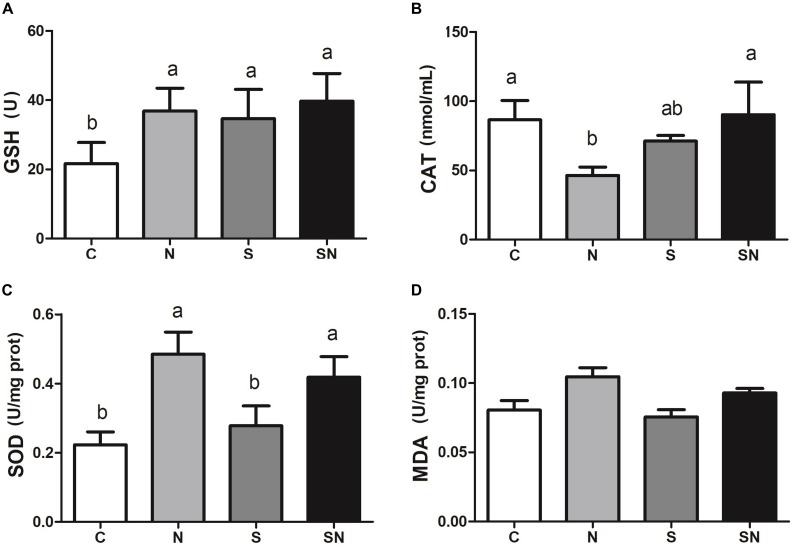
Effect of Fe_3_O_4_-NPs on GSH-Px **(A)**, CAT **(B)**, SOD **(C)**, and MDA **(D)** enzyme activities in the livers of chickens exposed to Fe_3_O_4_-NPs in the absence or presence of *S.* Enteritidis administration. Values represent the mean ±SEM. Different letters indicate statistically significant differences.

The results for oxidative related enzyme activities are shown in Figure 5. Fe_3_O_4_-NPs exposure significantly increased the GSH-Px and SOD contents and decreased CAT content relative to the control group. *S*. Enteritidis infection remarkably increased GSH-Px content relative to the control group. On the other hand, pretreatment with Fe_3_O_4_-NPs followed by *S*. Enteritidis infection (group SN) showed an obvious increase in SOD content relative to the *S*. Enteritidis infection group (group S) and control group (group C). No significant difference in MDA content was found in the 4 groups.

### *S.* Enteritidis Infection Induced Autophagy

**FIGURE 6:**
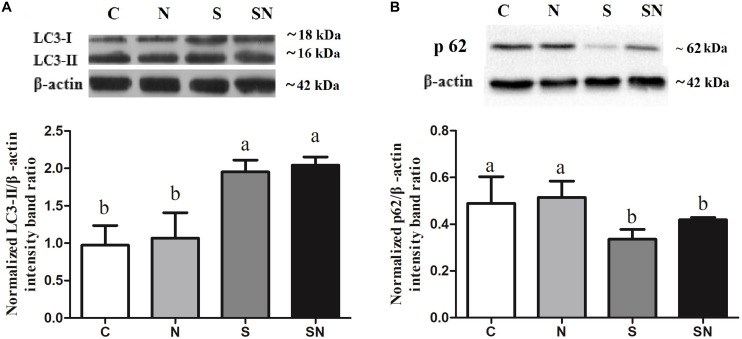
Effect of Fe_3_O_4_-NPs on LC3 **(A)** and p62 **(B)** protein expression in the livers of chickens exposed to Fe_3_O_4_-NPs in the absence or presence of *S*. Enteritidis administration. Values represent the mean ±SEM. Different letters indicate statistically significant differences.

Autophagy related protein LC3 and p62 expression in the 4 groups is shown in Figures 6A,B. LC3-II protein levels in group N did not change compared to those in group C, but significantly increased in groups SE and SN. p62 protein levels in group N did not change compared to those in group C, but significantly decreased in groups SE and SN. The Fe_3_O_4_-NPs exposure alone had no significant effect on LC3 and p62 protein levels compared with those of control.

### *S.* Enteritidis Induced Inflammation, Which Was Attenuated by Fe_3_O_4_-NPs

**FIGURE 7:**
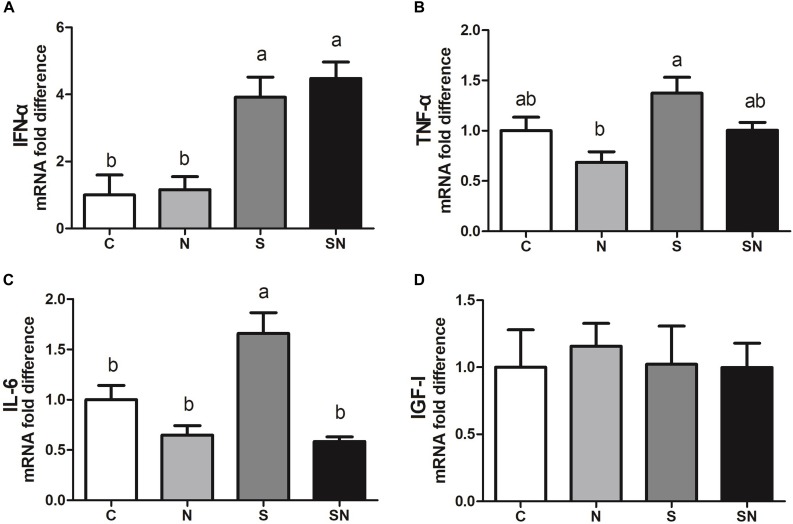
Effect of Fe_3_O_4_-NPs on IFN-α **(A)**, TNF-α **(B)**, IL-6 **(C)** and IGF-I **(D)** mRNA expression in the livers of chickens exposed to Fe_3_O_4_-NPs in the absence or presence of *S*. Enteritidis administration. Values represent the mean ±SEM. Different letters indicate statistically significant differences.

The results of inflammation factor analysis are shown in Figure 7. *S.* Enteritidis infection significantly increased IFN-α and IL-6 expression in the liver (group S) relative to control group. Fe_3_O_4_-NPs exposure (group N) showed no significant difference relative to the control group. Pretreatment with Fe_3_O_4_-NPs followed by *S.* Enteritidis infection (group SN) significantly decreased IL-6 expression compared that in the with *S*. Enteritidis infection group (group S). No significant difference was found in IGF-1 expression in four groups.

### *S.* Enteritidis Regulated PI3K/Akt/mTOR Signaling, Which Was Retroregulated by Fe_3_O_4_-NPs

**FIGURE 8:**
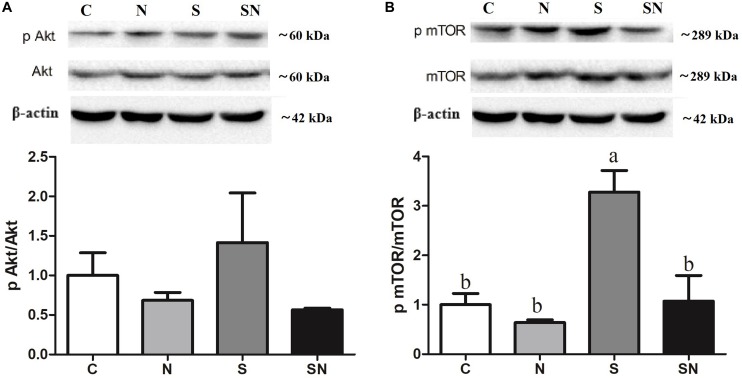
Effect of Fe_3_O_4_-NPs on phosphorylated protein expression in PI3K/Akt/mTOR signaling in the livers of chickens exposed to Fe_3_O_4_-NPs in the absence or presence of *S.* Enteritidis administration. **(A)** Phosphorylated Akt/Akt expression, **(B)** phosphorylated mTOR/mTOR expression. Values represent the mean ±SEM. Different letters indicate statistically significant differences.

Signaling pathway results are shown in Figure 8. As the key regulator of ROS and autophagy, we examined the phosphorylation level of Akt, and mTOR following different treatments. mTOR phosphorylation increased significantly in *S*. Enteritidis infected chickens (group S). Fe_3_O_4_-NPs pretreatment (group SN) markedly attenuated phosphorylation in the PI3K/Akt/mTOR signaling pathway.

## Discussion

Fe_3_O_4_ magnetic nanoparticles have received enormous attention for the creation of novel methods in biotechnology areas for their innovative properties in physical and chemical function. Most of the studies reported before were focused on the exploration of suitable dose, exposure routine and the organ pathology difference induced by Fe_3_O_4_-NPs (Mohanraj and Chen, 2007). Meanwhile, the bacterial inhibitory effect of Fe_3_O_4_-NPs was also studied and validated many years ago (Zhang et al., 2013; Sun et al., 2014). As a large proportion of studies reported were conducted *in vitro*, our previous research found that Fe_3_O_4_-NPs could effectively reduce the viability of intracellular *S*. Enteritidis in LMH cells (Shi et al., 2018). All of the above studies *in vitro* provided a new sense of hope for exploring novel methods of bacterial inhibition *in vivo*. In the poultry industry, there are growing calls to explore antibiotic alternatives in the control of bacterial infections. To our delight, the results of this trial confirmed that the oral administration of Fe_3_O_4_-NPs could significantly decreased the invasion of *S.* Enteritidis in chickens. With regard to the ROS generated by Fe_3_O_4_-NPs *in vivo* and *in vitro*, the exact mechanisms of the inhibition process have not been clearly defined. To further evaluate the effect of Fe_3_O_4_-NPs in the inhibition process, the oxidation state and immune response were detected in the present study, including the autophagy and signaling mechanism involved.

As a typical metal oxide nanozyme, Fe_3_O_4_-NPs demonstrate peroxidase-like activity or catalase-like activity in the mediums of different pH values (Wei and Wang, 2013). The peroxidase-like activity of the Fe_3_O_4_-NPs used in this study were validated in previous research (Fan et al., 2018). Meanwhile, the bacterial inhibitory effect of Fe_3_O_4_-NPs in the mediums at acidic pH was reported and the mechanisms was the degradation effect of Fe_3_O_4_-NPs in H_2_O_2_ based on the ROS that generated in the process (Gao et al., 2007). As the key proteins in the formation of autophagosome, LC3-II and p62 are usually used as the marker for the autophagy process (Kabeya et al., 2000). Our results in this study showed that *S.* Enteritidis infections significantly increased autophagy activity, while Fe_3_O_4_-NPs did not. Giving that autophagosomes are acid vacuoles, we presumed that Fe_3_O_4_-NPs possess peroxidase-like activity in the inhibition process. Results of this study showed that Fe_3_O_4_-NPs exposure increased the ROS level in the liver. The cytotoxicity of nanoparticle that reported in previous were considered to be relevant for the oxidative stress that mediated by ROS (Alarif et al., 2014; Sadeghnia et al., 2015). Considering the potential oxidative stress that caused by Fe_3_O_4_-NPs exposure, the activities of the antioxidant enzymes SOD and CAT, the levels of the non-enzymatic antioxidant molecule GSH-Px and the lipid peroxidation enzyme MDA were detected. The level of SOD and GSH-Px activity represent the defensive capabilities of the systems against the attack of free radical. The level of MDA activity represents the degree of lipid peroxidation. Our study confirmed that Fe_3_O_4_-NPs exposure significantly increased GSH-Px and SOD levels. As efficient free radical scavengers, the elevated levels of these two enzymes revealed the redox balance in this system. Meanwhile, the imbalance between ROS and antioxidants that represented in the process of oxidative stress has resulted in human diseases. All of these results indicated that the suppressive effect of Fe_3_O_4_-NPs on *S*. Enteritidis was related to the strengthen defense of the antioxidant system that mediated by oxidative stress. To be noted, some reports showed that the augmentation of MDA level in the process of oxidative stress resulted in cytotoxicity (Guo et al., 2016). In this study, MDA levels showed no significant difference in Fe_3_O_4_-NPs exposure groups, indicating that antioxidant defense mechanisms were normal in this situation. All of the above results indicated that higher ROS generated by Fe_3_O_4_-NPs exposure were favorable in the *S*. Enteritidis inhibition process. Autophagy and ROS were combined in the inhibition process, while a balance was maintained between the oxidative system and anti-oxidative system in the chickens. To further validate the speculation, inflammation factors and pathological changes were measured in this study.

To date, very limited data suggesting an interaction between the immune system and Fe_3_O_4_-NPs exposure is available (Patil et al., 2018). To study the anti-inflammatory response upon Fe_3_O_4_-NPs exposure in chickens, the immune response of chickens, including pro-inflammatory cytokines and chemokines, was also assayed. As a pivotal part of the innate immune system, IFN-α is induced when an organism is invaded. TNF-α is one of the cytokines induced by activated macrophages, which are stimulated by elevated IFN-α content. The content of TNF-α is reported as the pivotal regulator in the reaction systems that stimulated by pathogens (Li et al., 2012). IL-6, one of the interleukins in organisms, can stimulate cell proliferation involved in the immune response. In the process of inflammation maintain, cellular debris elimination and other immune attraction, the indicators assayed above were all involved (Kaiser et al., 2004). In the present study, *S*. Enteritidis infection significantly increased IFN-α, TNF-α, and IL-6 contents, which is consistent with previous studies (Gou et al., 2015). Fe_3_O_4_-NPs exposure in normal chickens showed no significant effect on immunocyte stimulation. However, Fe_3_O_4_-NPs exposure in *S*. Enteritidis infected chickens alleviated the immune response. It was reported in previous that iron oxide nanoparticles treatment in the rats significantly decreased the expression of IFN-α, TNF-α, and IL-6 at the inflammatory site (Shen et al., 2012; Calero et al., 2014). This may explain the alleviating effect of Fe_3_O_4_-NPs exposure in chickens. Meanwhile, livers of chickens infected with *S*. Enteritidis showed more vacuoles with widened interstitial space compared with birds in the other groups. Fe_3_O_4_-NPs pretreatment alleviated these inflammation and pathological changes resulted from *S.* Enteritidis infection. These results help to validate the alleviation effect of Fe_3_O_4_-NPs exposure in chickens.

As the key regulator pathway in the process of ROS and autophagy, we examined the AKT phosphorylation at Ser473 and mTOR phosphorylation at Ser2448 following different treatments. As a serine/threonine protein kinase in the cell signaling network, the phosphorylation of mTOR shows pivotal effect in the signal deliver system (Jiang et al., 2014). In the present study, mTOR phosphorylation increased significantly in *S*. Enteritidis infected chickens. It was reported that the inhibition of mTOR in cells resulted in the promotion of catalytic activity and induces autophagy (Singh et al., 2012). Thus, the expression of target phosphorylation proteins in PI3K/Akt signaling pathway can negatively influence the formation of autophagosome (Maiuri et al., 2007). As livers were collected 48 h post infection, we hypothesize that significantly increased mTOR phosphorylation may be part of the negative feedback regulating process in chickens to the increased autophagy induced by *S*. Enteritidis infections. Meanwhile, it was reported in previous research that autophagy can be activated in dependent of ROS through the PI3K/AKT/mTOR signaling pathway (Mi et al., 2016; Jiang et al., 2017). Results of this study revealed that Fe_3_O_4_-NPs exposure in normal chickens showed no significant effect on the PI3K/AKT/mTOR signaling. Combined with the ROS and autophagy results in this group, we speculate that ROS generation induced by the catalase-like activity of Fe_3_O_4_-NPs in chickens cannot activate autophagy through the PI3K/AKT/mTOR signaling pathway. However, Fe_3_O_4_-NPs exposure significantly decreased mTOR phosphorylation in *S*. Enteritidis infected chickens. This result may be related to the significant reduction of *S*. Enteritidis in chicken livers.

In summary, the present study indicated that Fe_3_O_4_-NPs pretreatment can effectively prevent the invasion of *S.* Enteritidis in chickens, including the attenuation of pathological changes and inflammation response caused by *S.* Enteritidis infection. Balance was maintained between the ROS and antioxidant systems during the process of *S*. Enteritidis inhibition. The PI3K/Akt/mTOR signaling pathways were involved in this process. Taken together, our research demonstrates that the bacteriostatic activity of Fe_3_O_4_-NPs in chickens is avaliable and safe, which can be an alternative to antibiotics for bacterial inhibition in poultry industry.

## Data Availability Statement

All datasets generated for this study are included in the article/Supplementary Material.

## Ethics Statement

The animal study was reviewed and approved by the Animal Care and Use Committee of the Poultry Institute, Chinese Academy of Agriculture Science.

## Author Contributions

SS and YS conceived and performed the experiment. SS and LG provided valuable guidance on the revision of the manuscript. YX, SZ, and SW acquired the data. YS analyzed and interpreted the data.

## Conflict of Interest

The authors declare that the research was conducted in the absence of any commercial or financial relationships that could be construed as a potential conflict of interest.

## Supplementary Material

FIGURE S1 Bacterial inhibition rates of Fe_3_O_4_-NPs at different dose in livers of *S*. Enteritidis infected chickens. SPF chicks with similar initial body weights were distributed into three groups (*n* = 6 each group): (1) control (denoted as 0), (2) 50 mg/kg Fe_3_O_4_-NPs (denoted as 50) and (3) 200 mg/kg Fe_3_O_4_-NPs (denoted as 200). All birds were administered with 0.1 mL of *S*. Enteritidis (1 × 10^8^CFU/mL) by injection. Values represent the mean ± SEM. ^∗^Indicates statistically significant difference.

FIGURE S2 Representative TEM images of Fe_3_O_4_-NPs in liver in Fe_3_O_4_-NPs treatment groups (scale bar = 0.5 μm).

## Abbreviations

Akt: protein kinase B
CAT: catalase
Fe_3_O_4_-NPs: Fe_3_O_4_ magnetic nanoparticles
GSH-Px: glutathione peroxidase
IFN- α: interferon- α
IGF-I: insulin-like growth factor 1
IL-6: interleukin-6
LC3: microtubule-associated protein light chain 3
MDA: malondialdehyde
mTOR: mammalian target of rapamycin
p Akt: phosphorylation Akt at Ser 473
p mTOR: phosphorylation mTOR at Ser 2448
p62: sequestosome 1
*S*. Enteritidis: *Salmonella* Enteritidis
SEM: scanning electronic microscopy
SOD: superoxide dismutase
TEM: transmission electron microscopy
TNF- α: tumor necrosis factor
