# Supplementary figures and images for: Fe3O4 Nanoparticles Attenuated Salmonella Infection in Chicken Liver Through Reactive Oxygen and Autophagy via PI3K/Akt/mTOR Signaling
Source: Front Physiol. 2020 Jan 17;10:1580. doi: 10.3389/fphys.2019.01580 (PMC6978669; doi:10.3389/fphys.2019.01580)

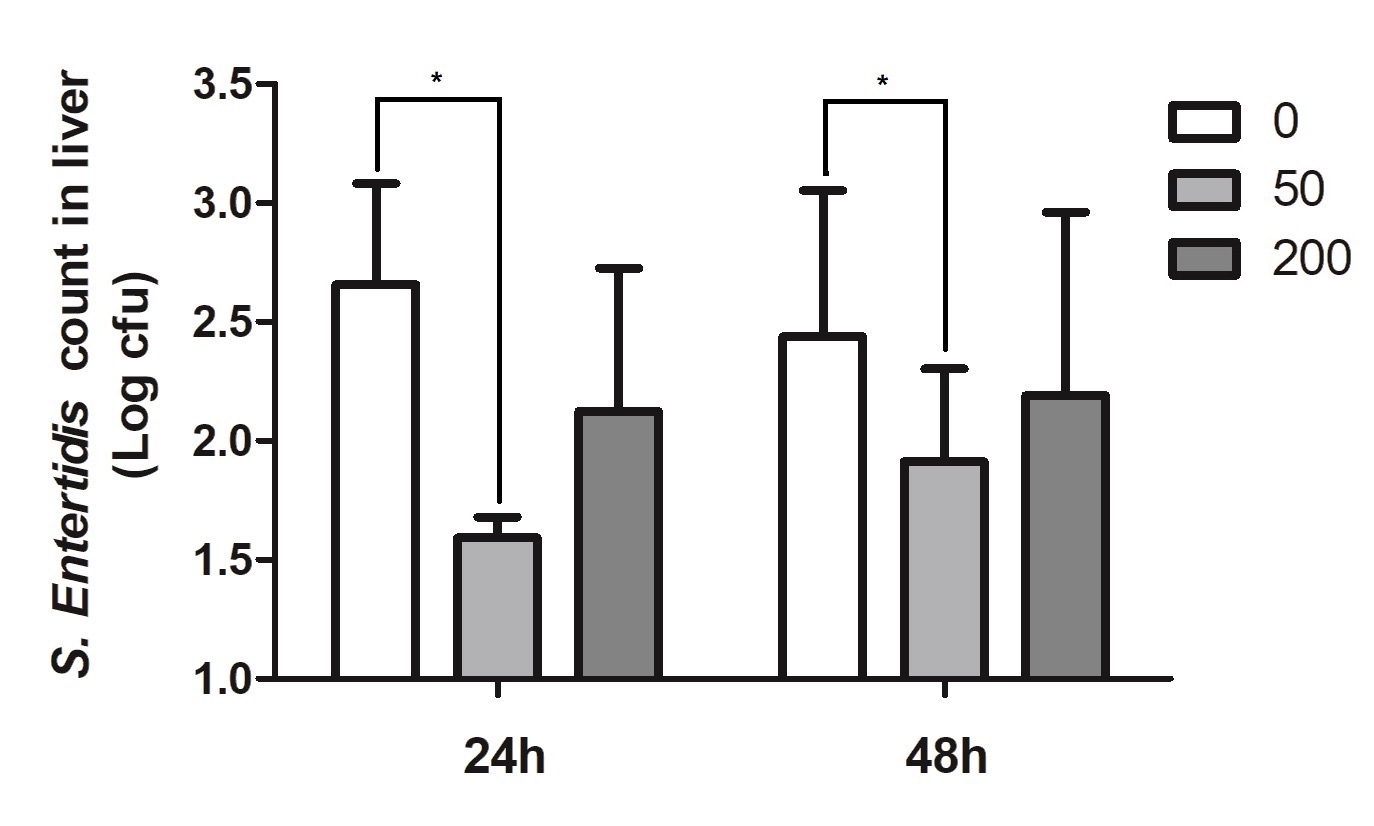

Supplement: FIGURE S1 — Bacterial inhibition rates of Fe3O4-NPs at different dose in livers of S. Enteritidis infected chickens. SPF chicks with similar initial body weights were distributed into three groups (n = 6 each group): (1) control (denoted as 0), (2) 50 mg/kg Fe3O4-NPs (denoted as 50) and (3) 200 mg/kg Fe3O4-NPs (denoted as 200). All birds were administered with 0.1 mL of S. Enteritidis (1 × 10^8^CFU/mL) by injection. Values represent the mean ± SEM. ∗Indicates statistically significant difference. [file Image_1.JPEG]

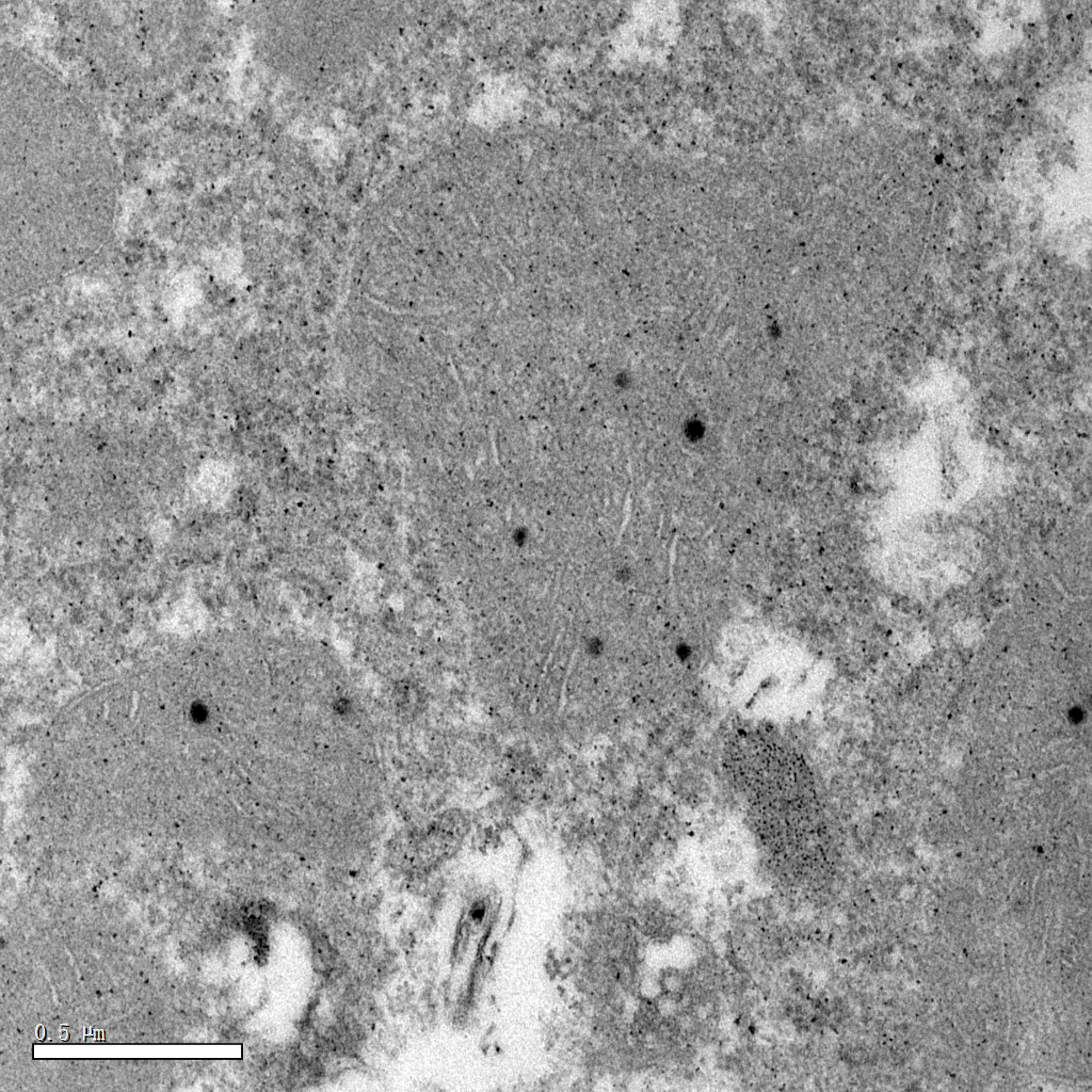

Supplement: FIGURE S2 — Representative TEM images of Fe3O4-NPs in liver in Fe3O4-NPs treatment groups (scale bar = 0.5 μm). [file Image_2.TIF]
